# Rural households vulnerability to covariate and idiosyncratic shocks using multilevel longitudinal model: evidence from Ethiopia

**DOI:** 10.1016/j.heliyon.2022.e10968

**Published:** 2022-10-08

**Authors:** Yalfal Temesgen, Mengistu Ketema, Alelign Ademe

**Affiliations:** a School of Agricultural Economics and Agribusiness, Haramaya University, Ethiopia; b Ethiopian Economic Association, Ethiopia; c Department of Agricultural Economics and Manag’t, University of Eswatini, Lesotho

**Keywords:** Vulnerability, Shocks, Panel data, Mixed effect, Ethiopia

## Abstract

The economic reports on income inequality, poverty, and other welfare indicators are relevant if it was supported by the evidence of income traps because rural households in developing countries were frequently hit by several shocks. This study intended to investigate the households' vulnerability to covariate and idiosyncratic shocks in Ethiopia using three waves of the household panel survey for the period of 2011–2016. The study applied a three-level mixed effect model for analysis. The result of the three-level mixed effect model points out that both covariate and idiosyncratic shocks) were found to influence household consumption and covariate shocks are a relatively larger and more significant effect on households' consumption than idiosyncratic shocks. Based on the finding of this study, it is suggested that the implementation of different policies that address factors contributing vulnerability to covariate and idiosyncratic shocks of rural households in Ethiopia.

## 1 Introduction

Measuring household welfare based on income, consumption, asset, and other indicators was gaining increasing importance in the economics literature. However, a study by Sen (1997) stated that the welfare difference mainly depends on the ability to absorb shocks occurred during course of action. A broad grasp of the current state of welfare is insufficient without considering the current state of households' exposure to various shocks (Wassie, 2019; Dercon, 2006; Krätli et al., 2013; Calvo and Dercon, 2013; Klasen et al., 2015). While it is becoming increasingly obvious that measures to reduce household vulnerability must be a cornerstone of any welfare improvement plan (World bank, 2015), the quantitative links between shocks and welfare are little established. This exposure to shocks defines as vulnerability, having a probability of suffering a future shortfall where vulnerable households are those with low opportunities and encounter difficulties when exposed to shocks (Asfaw et al., 2012; Lovo and Veronesi, 2019; Hassen et al., 2017; Singh et al., 2016; Elbers et al., 2003).

Studies of shocks are inextricably tied to vulnerability research. They seek to identify the systems, elements of systems, or people groups that are most vulnerable to the effects of a severe disturbance. These methods have resulted in the identification of vulnerable systems or vulnerable populations to implement activities in a prevention policy that will lessen the effects of shocks on the elements targeted. The theoretical bases of vulnerability are argued based on two perspectives; from broad perspective, a number of studies have attempted to classify the idea of vulnerability based on four basic approaches: the risk hazard approach, the political economics method, the coupled vulnerability approach, and the poverty viewpoint. From specific perspectives, the vulnerability assessment should answer issues like the extent of the vulnerability, the sources of vulnerability, and households' response to shocks using approaches including vulnerability measurement based on poverty, index-based, proxy means testing, expects utility and uninsured exposure to risk.

Rural households in Ethiopia are engaged in farming as the main sources of income; though, their income is an unstable duo to change in weather conditions, pest infestation, drought, market forces and the like. The exposure to shock is not only reducing income but also leads to the loss of critical, productive assets. A household that is not poor at present may face the risk of becoming poor in the future due to exposure to different shocks including covariate shocks, events that occurred among living within the same localities and idiosyncratic shocks, events that occurred for only within households or persons (Chaudhuri, 2002; Hoddinott and Quisumbing, 2003; Wossen et al., 2014; Seipt et al., 2013; Dercon, 2005; Krätli et al., 2013; Calvo and Dercon, 2013; Klasen et al., 2015; Wassie, 2019).

Although there has been a recent growing interest in the area of vulnerability, relevant empirical studies on vulnerability by decomposing shocks into covariate and idiosyncratic shocks still because, the majority of the studies are constrained by limited information on a full range of shocks (Tesfaye et al., 2015; Günther and Harttgen, 2009; Hoddinott, 2006; Dercon et al., 2005; Christiaensen, 2000; Chaudhuri, 2003; Ligon and Schechter, 2002). This is because empirical studies are still dominated on the vulnerability to poverty and climate change. One of the main reasons for this is the lack of nationally representative data in Ethiopia and limited scope in incorporating types of shocks in the study such as taking into account individual as well as community-level shocks (Dercon and Krishnan, 2000; Dercon, 2005). In light of this, our study made an effort to close the gap by conducting a vulnerability analysis (VA). The ability to identify (I) the vulnerable and (ii) the sources of vulnerability substantially improves the formulation of preventative welfare strategies.

Several empirical working in Ethiopia has focused on households' income and their vulnerability to poverty and climate change with static or snapshot evidence (Deressa et al., 2008; Tessema and Simane, 2019; Bedeke et al., 2020; Demeke et al., 2011; Bogale, 2012; Gelaw and Sileshi, 2013; Debebe and Zekarias, 2020). This study, however, provides vulnerability to shocks, by decomposing the relative impact of covariate and idiosyncratic shocks using panel data an empirical application to rural households in Ethiopia. This study has the potential to provide important insight into understanding vulnerability to a wide variety of shocks, which are likely to increase rural households' vulnerability to shocks, holds a great promise for policy and program design that is ex-ant in nature. Having these concerns, this study intended to answers the following questions. Who are households vulnerable to idiosyncratic and covariate shocks in Ethiopia and which factor play role in explaining the vulnerability to idiosyncratic shocks and covariate shocks? Thus, the objective of this study was to assess the households' vulnerability to covariate and idiosyncratic shocks, specifically, to examine the determinants of households vulnerability to idiosyncratic and covariate shocks, to understand the profile of rural household vulnerability to idiosyncratic and covariate shocks on the household welfare and its impact on the welfare and to identify which shocks have more influence on the rural household vulnerability.

## 2 Methodology

### 2.1 Data sources, collection methods and type

This study employed panel data from the Living Standard Measurement Survey-Integrated Agricultural Survey (LSMS-ISA), which was conducted by the World Bank and the Ethiopian Central Statistics Agency (CSA). The first wave, second wave, and third wave of the survey were collected in 2011/2, 2013/4, and 2015/6, respectively. The panel dataset is a nationwide survey that was compiled using household multistage probability samples. Using a stratified random design, the study’s domains (regions, urban/rural) are first identified. Second, probability proportional to size was used to choose the enumeration areas (EAs). Thirdly, the country’s most recent population census is used to determine the size of the main sampling units (PSU), which are geographically specified area units. The final step is the systematic selection of the secondary sampling units (households) (Table 1).

**Table 1:** The sampling frame and sample distribution of the survey.

	Wave One				Wave Two					Wave Three			
	Rural		Small town		Rural		Small towns	Large towns		Rural	Small towns	Large towns	
	EAs	HHs	EAs	HHs	EAs		EAs	EAs	HHs	EAs	EAs	EAs	HHs
National	280	3466	43	503	280		42	73	5262	280	42	73	4954
Tigray	30	360	4	48	30		4	15	633	30	4	15	633
Afar	10	120	2	24	10		2	1	159	10	2	1	159
Amhara	61	728	11	127	61		10	15	1077	61	10	15	1077
Oromiya	55	656	11	125	55		10	20	1080	55	10	20	1080
Somali	20	237	3	36	20		3	3	321	20	3	3	321
Benishangul	10	120	1	12	10		1	0	132	10	1	0	132
SNNP	74	885	10	119	74		10	15	1233	74	10	15	1233
Gambella	10	120	1	12	10		1	1	147	10	1	1	147
Harari	10	120	0	0	10		1	3	177	10	1	3	177
Total Households		3969				5262				4954			

*Note:* HHs = households interviewed, EAs = Enumeration area numbers.

With a total attrition rate of 6.8, a total of 3969 households from wave one, 5262 households from wave two, and 4954 households from wave three were questioned. Large town samples were only taken in the second and third waves, and as the study is primarily on rural families, those samples were automatically omitted from the analysis. In addition, the study limits the sample size due to zero total consumption and missing consumption data (Table 2).

**Table 2:** Sample size and excluding criteria.

No.	Excluded the household due to	Wave-one		Wave-Two		Wave-Three	
		Total	Excluded	Total	Excluded	Total	Excluded
1	Full sample (14,185)	3969	-	5262	1486	4954	1255
2	Lost to Attrition	3969	270	3776	77	3699	-
3	Missing information on	3669	67	3699	145	3699	146
4	No food consumption reported	3632	70	3554	168	3553	164
5	Unbalanced sample (10,337)	3562	323	3386	147	3389	150
6. Balanced sample (9717)		3239		3239		3239	

*Note:* ∗missing information refers to the purchased items with no valid conversion factor to convert food items to monetary values.

### 2.2 Method of data analysis

The descriptive statistics and Econometric tools were used for analysis. The descriptive statistics was used to provide a picture of demographic, socio-economic, institutional, wellbeing dynamics of the sampled households. To examine the sources and level of vulnerability the study adopted the method from Mina and Katsushi (2016) which were extended from Günther and Harttgen (2009) and proposed by Chaudhuri (2002). Following Christian and Katsushi (2012, 2015), Günther and Harttgen (2009) and Chaudhuri (2002), the consumption generating process was stated as (Eq. (1)):

(1) $$\ln\;c_{n}=\beta_{oj}+\beta_{1j}x_{1ij}+\beta_{2j}x_{2ij}+e_{ij}$$

where ln *c*_*n*_ is *per capita* (log) consumption that serves as the indicator of household welfare. *X* is a set of the household as well as community characteristics such as sex, age, education, land size, livestock, asset, land size, extension and credit service, distance to market and main road. β is a vector of the parameter to be estimated and *e*_*i*_ is a random error term. The above equation gives the mean level of consumption of the household *i* in Woreda *j*. To determine the variance of consumption, community-level characteristics can be included as follows;

(2) $$\begin{array}{l} \beta_{oj}=Y_{00}+Y_{01}Z_{j}+u_{oj}\\ \beta_{1j}=Y_{10}+Y_{11}Z_{j}+u_{1j} \end{array}$$

The final form of the model can be modified based on the above equations (Eq. (2)) as follows;

(3) $$\ln\;C_{ij}=Y_{00}+Y_{10}Z_{j}+\left(Y_{10}+Y_{11}Z_{j}\right)x_{ij}+u_{0j}+u_{1j}+\varepsilon_{ij}$$

The right-hand side of the equation (Eq. (3)) that has three terms is the deterministic or fixed part whereas the left-hand side is the stochastic or random part of the equation.

This study applied a multilevel model for analysis where multilevel modelling is an appropriate approach if one wants to analyze hierarchically structured data with variable define at all levels of the hierarchy (Hox, 2000). When data contain variables measured at different levels, nesting of lower level units with higher level ones produces additional sources variation that violate the independence and homoscedasticity assumption. This is true with panel data, where random fluctuations can occur at repeated measurements leading to serially correlated errors (Gibbons et al., 2010). Using multilevel models also allows us to include error terms at each level, which makes it possible to track changes in variance at each level across models (Gelman, 2009). This method was applied using the following step:

Step one: Regress the log of consumption per adult equivalent of household *i* (*n* = 1... *i*) in Woreda *j* at time *t* using the above Eq. (3). The level is justified in the ways that one is nested in the other, as the result level-one is the time or wave indicated by *t* = 1, 2, 3; level two is households indicated by *n* = 1..., *i*, and level three is Woreda indicate by *j* = 1, ..., *i*. Step two: the above equation gives the mean level of consumption of the household i in Woreda *j*. To determine the variance of consumption, square the residual generated in the first step and use as a dependent variable as indicated in Eq. (4).

(4) $$\begin{array}{l} e_{ij}^{2}=\delta_{0}+\delta_{1}X_{ij}+\delta_{2}Z_{ij}+\delta_{3}X_{ij}Z_{j}\\ u_{ij}^{2}=\tau_{0}+\tau_{1}Z_{j}\\ {\left(e_{ij}+u_{oij}\right)}^{2}=\theta_{0}+\theta_{1}X_{ij}+\theta_{2}Z_{ij}+\theta_{3}X_{ij}Z_{j} \end{array}$$

Step Three: The expected log of consumption and it variance including covariate, idiosyncratic and total variance is determined with the parameters of Eq. (5).

(5) $$\begin{array}{l} \hat{E}\left[\ln\;C_{i}/x\right]={\hat{\beta }}_{0}+{\hat{\beta }}_{1}x_{i}\\ \hat{V}\left[\ln\;C_{i}/x\right]={\hat{\sigma }}_{ei}^{2}={\hat{\theta }}_{0}+{\hat{\theta }}_{1}x_{i} \end{array}$$

Step four: These estimates from step three can be used to determine the vulnerability of the household to covariate and idiosyncratic shocks as follows (Eq. (6)):

(6) $${\hat{V}}_{tij}=\hat{P}\left(\ln\;y_{tij}<\ln\;z|x_{tij}^{T}\right)=\Phi \left(\frac{\ln\;z-\ln\;{\hat{y}}_{tij}}{\sqrt{{\hat{\sigma }}_{tij}^{2}}}\right)$$

Where: Φ(⋅) denotes the cumulative density of the standard normal distribution; lnz is the log of the poverty threshold, z; $\ln\;{\hat{y}}_{tij}$ is log of per capita income of household predicted by Eq. (3); and $V_{t+k,ij}^{*}=1-\left[P\left(\ln\;y_{tij}>\ln\;y\right)\right]$ is the expected total variance of residuals in Eq. (4). Vulnerability estimation is also conducted separately for different components of variance in income, namely: idiosyncratic variances ${\hat{\sigma }}_{e_{tij}}^{2}$and ${\hat{\sigma }}_{u_{0ij}}^{2}$, and covariate variance ${\hat{\sigma }}_{v_{0j}}^{2}$. Step Five: The optimal or benchmark vulnerability threshold is determined based on empirical literature as a result the most common vulnerability threshold used was 0.5 (Pritchett et al., 2000a, Pritchett et al., 2000b; Guther and Harttegen, 2009; Christian and Katsushi, 2012), is adopted in this study within the three waves. Having this benchmark vulnerability threshold, the vulnerability threshold that determines the future non-vulnerable and vulnerable household is determined as follows (Eq. (7)):

(7) $$V_{t+k,ij}^{*}=1-\left[P\left(\ln\;y_{tij}>\ln\;y\right)\right]$$

where, $V_{t+k,ij}^{*}$ is vulnerability status of the households at time t, $P\left(\ln\;y_{tij}>\ln\;y\right)$ is a probability of having income above or below the consumption thresholds. Thus, the estimated vulnerability threshold at time t to fall below the poverty threshold at least once in the next 3 years is 0.2063 and this was consistent with the result of Christian and Katsushi (2015).

## 3 Results and discussion

### 3.1 Characteristics of sample households

Vulnerability to shocks can be attributed to a number of socioeconomic factors. These include household traits including sex, age, education, highest educational attainment in the home, total number of individuals in the household, and the proportion of dependent and employed persons in the household. Table 3 provided the summary statistics for household characteristics. The majority of the sample houses were male-headed families, with 24% of them having female heads. The average age of the sample households was 46 years, with lowest and maximum ages of 14 and 100 years, respectively. The mean age of the household was, however, 26 years old when the entire family was taken into account. A variety of socioeconomic factors play a role. This suggests that the household leader is younger than the family’s average age and that the majority of the household’s members are children. The sampled households' average literacy rate was 39%. The sampled households had a mean of six members and a dependency ratio of 1.3%, which may be viewed from both the labor availability and expenditure perspectives. The outcome also revealed that there were on average 1.4 more female adults than male adults in the home. The outcome also revealed that each household’s average age ranged from 17 to 100.

**Table 3:** Demographic characteristic of the sampled households.

Variable		Mean	Std. Dev.	Min	Max	Observations
1. Sex of the Household head	Overall	0.243594	0.429272	0	1	N = 9717
	Between		0.42417	0	1	n = 3239
	Within		0.066268	-0.42307	0.91026	T = 3
2. Age of the Household head	Overall	46.30998	15.41563	14	100	N = 9491
	Between		15.27201	15.66667	100	n = 3238
	Within		3.231546	15.80998	92.64331	T-bar = 2.93113
3. Mean age in the household	Overall	25.94219	12.44577	6.75	97	N = 9715
	Between		11.523	10.8537	91.33333	n = 3239
	Within		4.703391	-17.5023	64.80885	T-bar = 2.99938
4. Household head Literacy	Overall	0.393023	0.488447	0	1	N = 9717
	Between		0.433647	0	1	n = 3239
	Within		0.224877	-0.27364	1.059689	T = 3
5. Education level in the Household	Overall	1.932695	1.420675	0	35	N = 9717
	Between		1.095344	0	19.66667	n = 3239
	Within		0.904868	-13.734	23.9327	T = 3
6. Household Size	Overall	5.559226	2.520626	1	18	N = 9717
	Between		2.384203	1	15.66667	n = 3239
	Within		0.818721	-1.77411	11.55923	T = 3
7. Total Male Adults	Overall	1.325924	1.041383	0	9	N = 9717
	Between		0.929917	0	8.333333	n = 3239
	Within		0.468948	-2.00741	3.99259	T = 3
8. Total Female Adults	Overall	1.413914	0.877374	0	8	N = 9717
	Between		0.761022	0	5.666667	n = 3239
	Within		0.43675	-1.25275	3.747247	T = 3
9. Dependency Ratio	Overall	1.33378	1.082267	0	11	N = 9374
	Between		0.868704	0	6	n = 3181
	Within		0.653508	-2.49955	8.222668	T-bar = 2.94687

Table 4 provides a summary of household consumption and income statistics. The average household consumed significantly more food than non-food items. In addition to being greater on average than farm incomes, off-farm incomes also had bigger standard deviations. This suggests that household incomes included off-farm incomes as a significant portion. Compared to farm incomes, off-farm incomes were distributed more unevenly.

**Table 4:** Summary of consumption, income and credit use of the sampled households.

Variables	First Wave		Second Wave		Third Wave		Overall	
	mean	S.D	mean	S.D	Mean	S.D	mean	S.D
Consumption (Household)								
1. Food Consumption	4485.25	4970.85	3912.45	3138.14	4079.16	3412.32	4158.95	3931.30
2. Non-food Consumption	1057.09	4072.31	1107.12	1330.12	1107.36	1361.46	1090.52	2595.12
3. Total Consumption	5542.34	6672.47	5019.56	3809.06	3809.06	4165.18	5249.48	5049.97
Income (Household)								
1. Farm income	762.96	3784	2435.97	8739.17	2164.98	7965.61	1787.97	7204.74
2. Non-farm income	3853.01	24388.	3852.23	30	6539.17	69704.42	4748.14	43777.69
3. Total income	4615.96	24636.	6288.20	19148.17	8704.15	70196.61	6536.11	44378.88
Credit Use (1 if access,0 otherwise)		-	0.284	0.451	0.42	0.58	0.179	0.384

### 3.2 Socio-economic and institutional characteristics of the sample households

Household well-being is influenced by socioeconomic and institutional factors such as total land holding, cultivated land size, livestock ownership, and access to infrastructure. To determine the statistical difference between consumption quintiles, these socioeconomic and institutional characteristics were tested across consumption expenditure quintiles. The test was also carried out to determine which consumption quintile differed from others. As shown in Table 5, there is a statistically significant mean difference between consumption quintiles based on socioeconomic and infrastructure factors such as land size, cultivated land, livestock holding, distance to the nearest market, and zone capital.

**Table 5:** Test of equality of means of socio-economic and institutional characteristics.

Variables	Overall	Consumption Quintile					
		1st	2nd	3rd	4th	5th	F-stat
Land size (ha)	1.35	1.17	1.37	1.49	1.63	1.12	2.22∗
	(6.43)	(2.88)	(3.31)	(3.31)	(8.44)	(3.40)	
Cultivated land (ha)	1.391	1.18	1.38	1.55	1.67	1.16	2.34∗
	(6.30)	(2.83)	(3.24)	(10.45)	(7.85)	(2.18)	
Livestock Holding (TLU)	2.73	2.19	2.7	2.89	2.90	2.88	11.88∗∗∗
	(3.57)	(2.59)	(3.72)	(3.53)	(3.62)	(4.11)	
Distance to main road (km)	16.41	16.37	16.37	16.52	16.82	15.99	0.38
	(21.98)	(18.93)	(21.10)	(22.68)	(23.55)	(22.92)	
Distance to Pop. Center (km)	40.49	40.89	39.51	40.54	40.07	41.33	0.66
	(33.66)	(28.31)	(30.02)	(34.06)	(36.04)	(37.75)	
Distance from nearest Market (km)	66.37	84.26	65.28	61.76	61.96	60.62	73.47∗∗∗
	(50.55)	(59.70)	(46.66)	(46.96)	(948.41)	(47.01)	

*Note:* ∗∗∗0.01% significant level: standard Deviation in the parenthesis based on pooled mean difference; mean differences by each wave.

In the first, second, third, fourth, and fourth consumption quintiles, the mean land size of the sampled households was 1.17, 1.37, 1.49, 1.63, and 1.12 ha, respectively, which is comparable to the national average of about 1.17 ha (Holden and Tilahun, 2020). According to the Tukey post hoc test, there is a statistically significant difference in mean land size and cultivated land between the fourth and fifth consumption quintiles. In the first, second, third, fourth, and fourth consumption quintiles, the sampled households owned 2.19, 2.7, 2.89, 2.9, and 2.88 TLU on average. The average household owned 2.73 TLU, which was lower than the national average. The post hoc test revealed a statistically significant difference in the mean.

The mean land size of the sampled household was 1.17, 1.37, 1.49, 1.63 and 1.12 ha in the first, second, third, fourth and fourth consumption quintiles, respectively and this is comparable to the national average of about 1.17 ha (Holden and Tilahun, 2020). The Tukey post hoc test indicates that there is a statistically significant difference in mean of land size and cultivated land between the 4th and 5th consumption quintiles. The sampled households owned 2.19, 2.7, 2.89, 2.9 and 2.88 TLU on average in the first, second, third, fourth and fourth consumption quintiles, respectively. Overall, households owned 2.73 TLU on average and this is lower than the national average. The post hoc test showed that there is a statistically significant difference in the mean of livestock holding between the 1th and other consumption quintiles.

### 3.3 Effect of vulnerability to covariate and idiosyncratic shocks

Understanding the effect of covariate and idiosyncratic shocks is very pertinent for policymaking to address the issue of rural household vulnerability. In this regard, sex, age and literacy of the household, household size, land and livestock holding, credit use, distance to road and market, income from farm and off-farm, food and non-food expenditure, exposure to shock, asset holding, extension contact, main livelihood and irrigation access were hypothesized to affect household consumption, thereby vulnerability. The analysis was conducted using a three-level mixed effect model. Before estimating the model, the study conducted a diagnostic test to arrive at the estimated model using a likelihood test. First, the OLS regression was tested against the two-level model and the two-level model was preferred. Second, a two-level model was tested against a three-level model and the three-level model was preferred. Finally, the likelihood test allows the inclusion of random coefficient for the time variable both at Woreda level and household’s levels. In addition to the likelihood test, the study also tests the variance-covariance matrix, group-level error and fitted values. The result of the three-level mixed model showed that education status of household head, household size, livestock holding, asset holding, distance to the nearest road, distance to the nearest market, extension contact, main livelihood, covariate and idiosyncratic shocks are the main factor which influences household consumption.

The education status of the household head is positively determined by household consumption which enables households to have access to information, skill and practices. This highlights the significance of education; a finding is consistent with the finding of Abrham and Bauer (2012), Birhan and Tesfahun (2017), Teka et al. (2019) and Kebede et al. (2019). Total household size is negatively determined household consumption as a larger household size means lower consumption. This implies that as the household size increase, its consumption declines. This is due to fact that larger household size is associated with higher sharing of resources. The result is in line with the finding from Lanjouw and Ravallion (1995), Bogale and Shimelis (2009) and Bersisa and Heshmati (2016).

Livestock holding is an important determinant of households' consumption as livestock holding increase the probability of households' consumption will raise. Gemechu et al. (2016), Abafit and Kim (2014) and Mitiku et al. (2012) showed that a household having higher livestock ownership creates more livestock products such as milk, milk products, meat, and better income from the sale of both live animal and livestock product. This highlights the positive effect of livestock on the household’s consumption; hence, this household has the potential of less vulnerable to shocks due to the consumption smoothing role of livestock. Similarly, asset holding is an important determinant of household consumption. The asset considered in this category is plow full sets, horse/donkey carts, wheelbarrows, boreholes, spray pumps, brewing trough, distilling equipment, fishnets, diesel pumps, water tanks, beehives, trailers, grinders, ax, slathers, hand hoes, spades, storage facilities, water tanks, bicycles, and radios.

The study found asset holding has a positive effect on the households' consumption. This result is confirmed with the previous studies (Woldeyohanes et al., 2017; Bachewe et al., 2016; Berloffa and Modena, 2013). The study also revealed that distance to the nearest market negatively determines the household’s consumption. It is obvious that when the distance to the nearest market decreases, the household’s access to price information will increase which discourages the market orientation behavior of the households. This is because the distance to the nearest market affects a household’s decision to engage in crop and livestock sales. This result is in line with the previous studies of Yonatan (2020) and Gemechu (2018). The agricultural extension contact has a positive effect on household consumption as the agricultural extension contact increases the households' access to agricultural practices, methods and information will be raised. This has a positive contribution to agricultural production and hence a positive effect on household consumption. This result is confirmed with the previous studies (Nkegbe et al., 2018; Hussein and Janekarnkij, 2013; Nugusse et al., 2013; Gregory and Sewando, 2013). The households' consumption will improve through promoting non-farm and off-farm rural livelihood diversification strategies (Dinku, 2018; Andualem et al., 2021).

The main livelihood negatively influences household consumption. This implies that the household who are mainly crop-based livelihood has lower consumption than other based livelihoods such as livestock, non-farm. This is because diversified livelihood enables households to have better income, enhance better consumption; this finding is consistent with the finding of Davis et al., 2014; Loison, 2015; Udoh and Nwibo, 2017; Gebru et al. (2018). The result also showed that both shocks (covariate and idiosyncratic) were found to have a negative influence on household consumption. Households that are less exposed to shocks have higher consumption than exposed ones. The study also found that covariate shocks are a relatively larger and more significant effect on households' consumption than idiosyncratic shocks. This result is consistent with the findings of Carter (1997), Dercon and Krishnan (2000), and Sarris and Karfkis (2010).

A recent study in Ethiopia also showed that covariate and idiosyncratic shocks have the highest share of household’s vulnerability to poverty and covariate shocks play a larger relative role in drought-prone lowland areas (Skoufias et al., 2021). This is because idiosyncratic shocks affect only the members of an individual household as opposed to covariate shocks, which involve entire communities (Frankenberger and Nelson, 2013). Rural households in Ethiopia as a culture of supporting each other during a difficult time for idiosyncratic shocks, relatively covariate shocks have a higher impact.

### 3.4 Vulnerability status and income dynamics of the households

The results of the vulnerability profile were presented in Table 6 where this result was computed based on the estimated three-level mixed model, income mobility classification, and operation assessment of vulnerability stated under Eq. (7). The result revealed that 25.65 percent of the households were classified as vulnerable were approximately 20 percent of the household were highly vulnerable and 6 percent of the households were moderately vulnerable. In addition, 18.82 and 10.28 percent of the households were vulnerable to unobserved covariate and idiosyncratic shocks, respectively. This result is consistent with the result already indicated in Table 7 above that covariate shocks have a slightly higher influence than idiosyncratic shocks on household consumption. This is because covariate shocks were more uncontrolled, not specific as well as direct to individual households. A study by Catherine (2008) indicated that households can smooth consumption when idiosyncratic shocks hit but appear not to smooth consumption during covariate shocks.

**Table 6:** Vulnerability profile of the household by income mobility status.

Vulnerability Status	Income mobility Status of the households			
	Downward	Immobility	Upward	Total
Based on fixed effect				
Not Vulnerable	3.08	1.93	2.13	7.13
Moderately Vulnerable	37.63	26.47	25.82	89.92
Highly Vulnerable	0.95	1.24	0.75	2.95
Based on the Random effect				
Total shocks				
Not Vulnerable	31.05	21.40	21.89	74.35
Moderately Vulnerable	2.75	1.90	1.34	5.99
Highly Vulnerable	7.85	6.35	5.46	19.67
Covariate shocks				
Not Vulnerable	34.16	23.56	23.46	81.18
Moderately Vulnerable	3.47	2.23	2.55	8.25
Highly Vulnerable	4.02	3.86	2.68	10.57
Idiosyncratic shocks				
Not Vulnerable	37.37	26.34	26.01	89.73
Moderately Vulnerable	3.27	2.23	1.80	7.30
Highly Vulnerable	1.01	1.08	0.88	2.98

**Table 7:** Mixed-effects REML model results of determinants household consumption.

	Coef.		St.Err	t-value	P-value	[95%		C. Interval]	Sig
Sex of the household head	0.011		0.030	0.36	0.722	-0.048		0.069	
Age of the household head	0.001		0.001	0.83	0.405	-0.001		0.002	
Education Status	0.119		0.024	5.05	0.000	0.073		0.166	∗∗∗
Land size	0.004		0.004	1.19	0.236	-0.003		0.012	
Livestock holding	0.035		0.003	11.02	0.000	0.029		0.041	∗∗∗
Household size	-0.066		0.005	-12.85	0.000	-0.076		-0.056	∗∗∗
Distance to nearest Market	-0.003		0.000	-13.87	0.000	-0.004		-0.003	∗∗∗
Distance to major road	0.002		0.001	3.53	0.000	0.001		0.003	∗∗∗
Irrigated use	-0.002		0.004	-0.46	0.646	-0.011		0.007	
Assets	0.070		0.005	13.49	0.000	0.06		0.08	∗∗∗
Credit use	0.000		0.000	-0.24	0.81	000		0.000	
Extension contact	0.087		0.023	3.82	0.000	0.132		0.043	∗∗∗
Idiosyncratic shock	-0.055		0.028	-1.95	0.051	-0.111		0.000	∗
Covariate shock	-0.136		0.023	-6.01	0.000	-0.092		-0.181	∗∗∗
Dependency Ratio	0.000		0.001	0.71	0.479	-0.001		0.001	
Main livelihood	-0.127		0.044	-2.89	0.004	-0.214		-0.041	∗∗∗
Woreda Level									
Variance (Random Slope)		8.820	0.074	119.54	0.000		8.675	8.964	∗∗∗
Variance (Random Intercept)		-2.764	0.339	-8.15	0.000		-3.429	-2.10	∗∗∗
Variance (Slop, Intercept)		-2.962	1.075	-2.76	0.006		-5.069	-0.855	∗∗∗
Household Level									
Variance (Random Slope)		-3.886	0.076	-50.97	0.000		-4.036	-3.737	∗∗∗
Variance (Random Intercept)		-1.230	0.085	-14.43	0.000		-1.397	-1.063	∗∗∗
Variance (Slop, Intercept)		5.727	34.60	0.17	0.869		-62.091	73.544	
Wave Level									
Wave 1:Variance (Residual)		-0.640	0.055	-11.59	0.000		-0.749	-0.532	∗∗∗
Wave 2:Variance (Residual)		-0.167	0.065	-2.59	0.01		-0.294	-0.041	∗∗∗
Wave 3:Variance (Residual)		-0.133	0.082	-1.64	0.102		-0.293	0.026	
Number of obs		9717		Chi-square			804.462		
Prob > chi2		0.000		Akaike crit. (AIC)			5335.643		

*Note: ∗*0.1 and *∗∗* 0.05% and *∗∗∗*10% significant level, respectively: standard Deviation in the parenthesis based on pooled mean difference; mean differences by each wave.

Let’s now look at a more detailed classification of vulnerability; it can be observed that about 10.50 percent of downward income movers and 6. 80 percent of upward income movers were classified as vulnerable to both shocks. The result also showed that about 7.49 percent of downward income movers were vulnerable to unobserved covariate shocks and 5. 23 percent of upward income movers were vulnerable to unobserved covariate shocks. Likewise, about 4.28 percent of downward income movers and 2.68 percent of upward income movers were vulnerable to unobserved idiosyncratic shocks. The result is con consistent with the findings of Krebs et al. (2013) where shocks have a higher influence on the downward income movers.

The results of the fixed effect part presented in Table 5 did not take into account time-invariant covariates and unobserved heterogeneity effect at the Woreda level. As the result, the estimated fixed part is higher than the random part. This estimate revealed that the majority of the households (92.87%) were classified as vulnerable to covariate and idiosyncratic shocks. In terms of income mobility status, the vulnerability is higher in downward income mover (38.38%) than upward income movers (26.57). This result is consistent with the findings of Mina and Imai (2016) where almost all households are classified as vulnerable based on the fixed effect result.

## 4 Conclusions

A recent estimate of income inequality, poverty and other welfare indicator showed an improvement in Ethiopia. However, the observed households' income dynamics will be full-fledged if the investigation has incorporated the vulnerability of the household to covariate and idiosyncratic shows because rural households in Ethiopia are frequently hit by several shocks resulting in consumption instability. The overall objective of the study was to generate evidence on households' vulnerability to covariate and idiosyncratic shocks in Ethiopia.

This study used a balanced panel data collected by the World Bank in collaboration with Ethiopia Central Statistics Agency (CSA) as the Living Standard Measurement Survey-Integrated Agricultural Survey (LSMS-ISA) that were randomly drawn from nine regions through a multi-stage sampling method. The survey has three rounds collected in 2011/2, 2013/4 and 2015/6. The panel has a total of 3969, 5262 and 4954 households from one, two and three waves, respectively. However, the study restricted the sample size duo missing information and a total of 9717 households were used for analysis.

The three-level mixed effect model result showed that education status of household head, household size, livestock holding, asset holding, distance to the nearest road, distance to the nearest market, extension contact, main livelihood, covariate and idiosyncratic shocks are the main factor which influences household consumption. The result of the three-level mixed-effect model was also used to constructed the vulnerability of the household and the profile showed that both covariate and idiosyncratic have considerable influence on households vulnerability covariate shocks has relatively higher impact.

Having literate households' heads found to have higher consumption, hence less vulnerable than illiterate household heads because education has a better opportunity to have access to information, skill and practices. Therefore, it is important to promote and support education both formal and non-formal considering the situations of rural households.

Livestock plays an important role in rural households including sources of food, energy, income and generator of development in most cases. As the livestock holding increase, the households' source of income and consumption will also increase. Thus, policy should encourage the livestock sector to bring the required output. This can be done by improving access to facilities and institutions targeting livestock including market, health, feed and information.

Total household size has a significant and negative effect on household consumption. A household that has a larger household size has a lower probability of consumption, hence higher vulnerable. Therefore, it is very pertinent to promote, support and strength the family planning program as well as investigate the more detailed study on the merit and demerits of household size on income, consumption, and other welfare indicators.

The study also revealed that asset holding has a significant and positive effect on household consumption. The asset holding is linked mainly for agricultural production that will lead to taking a path for improving the income of the rural households. As the household’s asset holding increases, the household’s income sources, consumption smoothing and livelihood options will increase. Thus, policy should promote certain key assets that will influence the economic choice of rural households.

The finding also revealed that distance to the nearest market negatively influences the household’s consumption. Market distance has implications on the households' decision to participate in marketing due to its role in access price information and the cost of transportation. Therefore, it is vital to promote the establishment of market place considering the rural household’s situation, promote collective action such as cooperatives and support the market infrastructures.

Whereas, agricultural extension services were found to have a significant and positive effect on household consumption. It is believed that agricultural extension has a positive contribution to agricultural production and productivity due to its role in accessing agricultural practices, methods and information on agriculture-related activities. Therefore, it is recommended that the current extension system should be strengthened and supported by finance, training and monitoring.

The finding of this study also showed that crop-based main livelihood negatively influences household consumption. This is because non-diversified livelihood enables households to have lower income, enhance lower consumption. Therefore, it is important to promote and support households diversify income through non-farm and off-farm rural livelihood diversification strategies.

Regarding exposure to covariate and idiosyncratic shocks, the study found that it has a negative and significant effect on upward income mobility as well as on consumption. Rural households face several misfortunes where they are exposed to economic, social, environmental and political shocks that repeatedly affect agricultural production and economic activities which will reinforce expose downward income mobility.

Thus, rural household in Ethiopia has suffered from frequent covariate and idiosyncratic shocks. Through the implementation of a social protection strategy that may include shock prevention, ex ante social insurance, and ex post social assistance, the government and the international aid community can lessen the detrimental consequences of these shocks on the food security of vulnerables. Social protection enables disadvantaged groups to better manage their shocks and contributes to the connection between development and alleviation.

## Declarations

### Author contribution statement

Yalfal Temesgen: Analyzed and interpreted the data; Wrote the paper.

Mengistu ketema: Conceived and designed the experiments; Contributed reagents, materials, analysis tools or data.

Alelign Ademe: Contributed reagents, materials, analysis tools or data.

### Funding statement

Mr. Yalfal Temesgen Tigabu was supported by Africa Economic Research Consortium (AERC) (REF:PH/TH/21-016 (AWARD- 1759)).

### Data availability statement

Data associated with this study has been deposited at https://www.worldbank.org/en/programs/lsms.

### Declaration of interest’s statement

The authors declare no conflict of interest.

### Additional information

No additional information is available for this paper.
